# Improving RNA nearest neighbor parameters for helices by going beyond the two-state model

**DOI:** 10.1093/nar/gky270

**Published:** 2018-05-01

**Authors:** Aleksandar Spasic, Kyle D Berger, Jonathan L Chen, Matthew G Seetin, Douglas H Turner, David H Mathews

**Affiliations:** 1 Department of Biochemistry & Biophysics, University of Rochester Medical Center, Rochester, NY 14642, USA; 2 Center for RNA Biology, University of Rochester Medical Center, Rochester, NY 14642, USA; 3 Department of Chemistry, University of Rochester, Rochester, NY 14627, USA; 4 Department of Biostatistics & Computational Biology, University of Rochester Medical Center, Rochester, NY 14642, USA

## Abstract

RNA folding free energy change nearest neighbor parameters are widely used to predict folding stabilities of secondary structures. They were determined by linear regression to datasets of optical melting experiments on small model systems. Traditionally, the optical melting experiments are analyzed assuming a two-state model, i.e. a structure is either complete or denatured. Experimental evidence, however, shows that structures exist in an ensemble of conformations. Partition functions calculated with existing nearest neighbor parameters predict that secondary structures can be partially denatured, which also directly conflicts with the two-state model. Here, a new approach for determining RNA nearest neighbor parameters is presented. Available optical melting data for 34 Watson–Crick helices were fit directly to a partition function model that allows an ensemble of conformations. Fitting parameters were the enthalpy and entropy changes for helix initiation, terminal AU pairs, stacks of Watson–Crick pairs and disordered internal loops. The resulting set of nearest neighbor parameters shows a 38.5% improvement in the sum of residuals in fitting the experimental melting curves compared to the current literature set.

## INTRODUCTION

Folding free energy change nearest neighbor parameters are used to estimate the folding stability of nucleic acid secondary structures. For RNA, these parameters (1) are widely used in software for secondary structure prediction, siRNA design, and non-coding RNA discovery (2–6). For DNA, these parameters (7) are also widely used in primer and nanostructure design (8–10).

Martin *et al*. (11) first determined folding free energy changes for RNA with optical melting experiments. For Watson–Crick helices, it was shown that optical melting data could be predicted with reasonable accuracy using a nearest neighbor model where the free energy for forming a base pair depended only on the identities of the neighboring base pairs. This led to the first sets of nearest neighbor parameters (12–14). The model was then expanded with more terms (15) and more data for helices (16,17) as well as additional terms for estimating loop stabilities (18–21).

The nearest neighbor parameters were derived from optical melting experiments fit with a two-state assumption for the melting behavior. This assumes that the transition from folded to unfolded state is abrupt for a given helix, i.e. at any temperature, a nucleic acid molecule is either completely folded or a random coil. With this assumption, the enthalpy and entropy changes of the transition can be fit to a single melting curve with a non-linear fit (22,23). Given the results of these fits for multiple sequences, nearest neighbor parameters can be fit using linear regression (16).

One important use of nearest neighbor parameters is to compute the partition function and predict individual base pair probabilities for single-stranded (24,25) or multiple-stranded structures (26–30). The partition function description of nucleic acids, however, predicts a prominent non-two-state behavior, with fraying of terminal base pairs and opening of AU pairs, a behavior that is also observed in NMR experiments (31–34). This contradicts the assumption under which the nearest neighbors are derived, i.e. that molecules are either completely folded or unfolded. This creates a paradox where a method is using parameters to predict a behavior that is impossible under the assumptions with which the parameters were derived. In previous work (26), a partition function was used to predict melting temperatures and these were compared to the two-state fitted values. This showed prominent differences between the two models. Agreement with the optical melting curves, however, was not tested.

This work presents a method to determine a set of nearest neighbor parameters that properly describes the fact that RNAs can populate more than two conformations. Rather than using parameters derived with the two-state assumption, the partition function formalism was used to fit RNA nearest neighbor parameters directly to the melting experiments. The fraction of base pairing estimated with the partition function model was fit to the fraction of base pairing measured by optical melting experiments using a non-linear least-squares model. The fitting parameters are enthalpy and entropy changes for stacks of Watson–Crick pairs, intermolecular initiation, terminal AU pairs and disordered loops from size of two to sixteen nucleotides. Although the application of the new parameters is limited because only a subset of RNA optical melting experiments are currently available as the UV absorbance data, these new parameters describe the melting temperatures and curves better than parameters that relied on the two-state assumption. The new fitting approach also solves the paradox that experiments and calculations demonstrate non-two-state behavior in duplexes.

## MATERIALS AND METHODS

### Melting data

Thirty four unique duplexes were used in fitting. Each sequence had melting curves for multiple strand concentrations, for a total of 285 melting curves. Each melting curve had between 100 and 160 data points, giving a total of 39 382 points used in fitting. 20, 2, 6 and 1 sequences, respectively, were from the UV melting data of Xia *et al*. (16), Rauzan *et al*. (35), Kierzek *et al*. (36) and Nguyen *et al*. (37), and five additional duplexes were optically melted for this study. Table 1 lists all the duplexes and melts used in the fit. All duplexes had lengths from 5 to 10 base pairs and 11 duplexes were self-complementary. All optical melting experiments were performed in a buffer containing 1 M NaCl, 10 or 20 mM sodium cacodylate and 0.5 mM EDTA, pH 7. The results of the two-state fit for the duplexes melted in this work are in the Supplementary Data (Figure S1 and Table S1).

**Table 1.** List of unique duplexes used in the fit. The second column gives the number of melting curves that were present for each sequence and the third column gives the sequence. The slash symbol, ‘/’, next to the sequence denotes that the sequence is non-self-complementary. and paired with the fully complementary sequence. The fourth column gives the references for experimental data. For the last five sequences, which were melted in this work, the results of two state fits are given in the Supplementary Materials

Sequence #	# Melts	Sequence 5′ to 3′	Reference
1	9	GACUCAG/	(16)
2	12	GAGGAG/	(16)
3	7	GAGUGAG/	(16)
4	6	GCACG/	(16)
5	6	GCUCG/	(16)
6	9	GGCUUCAA/	(16)
7	9	GUCACUG/	(16)
8	8	GUGUCG/	(16)
9	7	UAUGCAUA	(16)
10	15	UCAGACU/	(16)
11	6	UCAUGA	(16)
12	5	UCGCU/	(16)
13	10	UCUAUAGA	(16)
14	9	UGACAGU/	(16)
15	13	UGAUCA	(16)
16	3	UGCGU/	(16)
17	9	UUCCGGAA	(16)
18	9	UUGCGCAA	(16)
19	7	UUGGCCAA	(16)
20	9	UUGUACAA	(16)
21	7	CAGUCAGU/	(36)
22	7	UCAAUUAGU/	(36)
23	9	UCACUGAGU/	(36)
24	11	UCAGUCAG/	(36)
25	8	UCAGUCAGU/	(36)
26	8	UCAUUAAGU/	(36)
27	7	CACAGCAC/	(35)
28	7	CACGGCUC/	(35)
29	8	UUAUCGAUAA/	(37)
30	11	AUCGGUA/	This work
31	9	UUGACCAU/	This work
32	6	AACUAGUU	This work
33	6	AGUAUACU	This work
34	13	UAGAUCUA	This work

Melting curves measure absorbance as a function of temperature, *A*(*T*). In this work, curves were transformed into fraction of maximal base pairs formed as a function of temperature using the following procedure for calculating thermodynamic parameters from melting data (38). Upper and lower baselines were linearly fit to get the upper and lower slopes *m_u_* and *m_l_* and intercepts *b_u_* and *b_l_*. Fitting was done with the linear least-squares method described in Press *et al*. (39). The number of points used to define lower and upper baselines was set to the first and last 10% of total number of points in each melting data set. The melting data were then plotted with both baselines and, if needed, the number of points was increased or decreased manually to ensure the baselines accurately describe upper and lower linear parts of the melting curves. The upper and lower baselines approximate the temperature dependent change in absorbance of the purely random coil and double helix conformations, respectively. The fraction of maximal pairs formed at temperature *T*, ${X _{melt}}(T)$, was then calculated as,

(1) \begin{equation*} {X _{melt}} (T) = \frac{{\left( {{m_{u\ }}T + {b_{u}}} \right) - A\left( T \right)}}{{\left( {{m_{u\ }}T + {b_{u}}} \right) - \left( {{m_{l\ }}T + {b_{l}}} \right)}}. \end{equation*}

Supplementary Figure S2 shows an example fit of the base lines and the resulting transformation from *A*(*T*) to *X*_melt_(*T*).

This procedure assumes that the relative hypochromicity of AU and GC pairs is the same. This assumption was tested by plotting the hypochromicities of sequences as a function of their ratio of AU to GC base pairs. Weak collinearity of the resulting plot indicates that the relative hypochromicities of AU and GC cannot be reliably determined from melting curves in the available dataset. The plot of this dependence and the accompanying calculations are in the Supplementary Material (Figure S3). It is possible, however, that the sequence dependence of the hypochromicity is a function of base pair stacking in addition to base pairing. A focused study that determined the sequence dependence of hypochromicity might be able to improve the accuracy of nearest neighbor parameter fits reported here.

Strand concentrations, ${C_T}$, were calculated for each optical melting experiment by estimating from UV absorbance at temperatures >80°C, and these values were used here.

### Calculating experimental melting temperature by linear interpolation

Experimental melting temperatures, ${T_m}$, were calculated directly from the melting curves, which, as described above, were represented as a fraction of maximal base pairs as a function of temperature, $X(T)$. The ${T_m}$ is defined as the temperature at which $X(T)$= 0.5, and this region of the melting curve is roughly linear. Therefore, ${T_m}$ was determined by linear interpolation between two points with ratios of double helices just above and just below 0.5, using the definition of a line. If $X({{T_1}})$ and $X({{T_2}})$ are the double helical ratios at temperatures ${T_1}$ and ${T_2}$ just above and just below $X ( {{T_m}} )$= 0.5, then:

(2) \begin{equation*}{T_m} = {T_1}\ + \left( {{T_2} - {T_1}} \right)\frac{{0.5 - X\left( {{T_1}} \right)}}{{X\left( {{T_2}} \right) - X\left( {{T_1}} \right)}}.\end{equation*}

### Estimating double helical fraction using the partition function

The fraction of paired nucleotides in a duplex was estimated with a partition function formalism. The partition function, $Q$, is calculated by summing the folding equilibrium constants of all possible secondary structures (24,25):

(3) \begin{equation*}Q = \sum\nolimits_i {exp} \,\left( { - \frac{{\Delta G_i^\circ}}{{RT}}} \right),\end{equation*}

where Δ*G°* is the folding free energy change, *R* is the gas constant and *T* is the absolute temperature in kelvins.

For simplicity, the partition function calculations assume that the strands are not allowed to slip, i.e. a base is only allowed to pair to its partner on the opposite strand. This is a reasonable assumption because the strands were designed to have only one possible pairing registration. Strands are allowed to form internal disordered loops of up to 8 × 8 nucleotides, because larger loops are not possible for sequences with a maximum of 10 bp. The partition function is used to calculate the probability of base pairs, ${P_{i,bp}}(T,{{\rm{\Delta }}{H_j},{\rm{\Delta }}{S_j}} ),$ which can then be directly compared to the experimentally determined fraction of base pairs. The fraction of maximal base pairs, $X ( {T,{\rm{\Delta }}{H_j},{\rm{\Delta }}{S_j}} ),$ at temperature, *T*, can be calculated from the probability of the base pair at position $i$, ${P_i}(T,{{\rm{\Delta }}{H_j},{\rm{\Delta }}{S_j}} )$:

(4) \begin{eqnarray*} &&X \ \left( {T,{\rm{\Delta }}{H_j},{\rm{\Delta }}{S_j}} \right) = \frac{{\mathop \sum \nolimits_{1 < i < L} {P_i}\left(T, {{\rm{\Delta }}{H_j},{\rm{\Delta }}{S_j}} \right)}}{L}\ \nonumber \\ &&= \frac{{{P_{bp}}\left( {T,{\rm{\Delta }}{H_j},{\rm{\Delta }}{S_j}} \right)\mathop \sum \nolimits_{1 < i < L} {P_{i,bp}}\left( {T,{\rm{\Delta }}{H_j},{\rm{\Delta }}{S_j}} \right)}}{L}\ , \end{eqnarray*}

where *L* is the length of the duplex (and the maximum number of possible pairs), and ${\rm{\Delta }}{H_j}$ and ${\rm{\Delta }}{S_j}$ are enthalpy and entropy changes of the nearest neighbor parameters, respectively. ${P_{bp}}( {T,{\rm{\Delta }}{H_j},{\rm{\Delta }}{S_j}} )$ is the probability that there is at least one pair formed in the helix. The probability that at least one pair is formed ${P_{bp}}( {T,{\rm{\Delta }}{H_j},{\rm{\Delta }}{S_j}} )$ for a non-self-complementary duplex (22,23,40,41) is

(5a) \begin{eqnarray*}&&{P_{bp}}\ \left( {T,{\rm{\Delta }}{H_j},{\rm{\Delta }}{S_j}} \right) \nonumber \\ &&{=} \frac{{1 {+} \left( {Q\left( {T,{\rm{\Delta }}{H_j},{\rm{\Delta }}{S_j}} \right) {-} 1} \right){C_T} {-} \sqrt {1 {+} 2\left( {Q\left( {T,{\rm{\Delta }}{H_j},{\rm{\Delta }}{S_j}} \right) {-} 1} \right){C_T}} }}{{(Q\left( {T,{\rm{\Delta }}{H_j},{\rm{\Delta }}{S_j}} \right) {-} 1){C_T}}}\ ,\end{eqnarray*}

The equivalent for self-complementary sequences, is

(5b) \begin{eqnarray*}&&{P_{bp}}\ \left( {T,{\rm{\Delta }}{H_j},{\rm{\Delta }}{S_j}} \right) \nonumber \\ &&{=} \frac{{1 {+} 4\left( {Q\left( {T,{\rm{\Delta }}{H_j},{\rm{\Delta }}{S_j}} \right) {-} 1} \right){C_T} {-} \sqrt {1 {+} 8(Q\left( {T,{\rm{\Delta }}{H_j},{\rm{\Delta }}{S_j}} \right) {-} 1){C_T}} }}{{4\left( {Q\left( {T,{\rm{\Delta }}{H_j},{\rm{\Delta }}{S_j}} \right) {-} 1} \right){C_T}}}\ ,\end{eqnarray*}

Here, $Q$ is the partition function calculated at temperature $T$ using the enthalpy and entropy changes of nearest neighbor parameters, ${\rm{\Delta }}{H_j}$ and ${\rm{\Delta }}{S_j}$ and ${C_T}$ is the strand concentration. One is subtracted from references to *Q*(*T*,Δ*H*_j_,Δ*S*_j_*)* to remove the unpaired state, and therefore *Q*(*T*,Δ*H*_j_,Δ*S*_j_*)* is the ensemble equilibrium constant to a state with at least one base pair. The probability that there is a base pair between nucleotide $i$ and its complement on the other strand, given that there is at least one pair, is calculated from the partition function as:

(6) \begin{eqnarray*}&&{P_{i,bp}}\ \left( {T,{\rm{\Delta }}{H_j},{\rm{\Delta }}{S_j}} \right) \nonumber \\ &&= \frac{{{Q_{L,i}}\left( {T,{\rm{\Delta }}{H_j},{\rm{\Delta }}{S_j}} \right){Q_{R,i}}\left( {T,{\rm{\Delta }}{H_j},{\rm{\Delta }}{S_j}} \right)}}{{Q\left( {T,{\rm{\Delta }}{H_j},{\rm{\Delta }}{S_j}} \right) - 1}}\ \nonumber \\ &&= \frac{{{Q_{L,i}}\left( {T,{\rm{\Delta }}{H_j},{\rm{\Delta }}{S_j}} \right){Q_{R,i}}\left( {T,{\rm{\Delta }}{H_j},{\rm{\Delta }}{S_j}} \right)}}{{\mathop \sum \nolimits_{1 \le k \le L} {Q_{L,k}}\left( {T,{\rm{\Delta }}{H_j},{\rm{\Delta }}{S_j}} \right)}}\ \end{eqnarray*}

where ${Q_{L,i}}$ and ${Q_{R,i}}$ are partial partition functions with a base pair at $i$ that includes all sequence and structures to the left (5′ direction) and to the right (3′ direction) from $i$, respectively. ${Q_{L,i}}$ and ${Q_{R,i}}$ are computed by recursion starting from 5′ and 3′ ends of sequence, respectively. The recursions for computing these quantities are given in the Supplementary Materials.

### Fitting procedure using partition function

A non-linear least squares fit was performed to minimize the residuals, i.e. the sum of squared difference between the measured and estimated fraction of maximal pairing. The enthalpy and entropy changes of nearest neighbors were the fitting parameters:

(7) \begin{equation*} {\rm{min}}\left[ {\sum {{\left( {{X _{melt}}(T) - X ( {T,{\rm{\Delta }}{H_j},{\rm{\Delta }}{S_j}})} \right)}^2}} \right] \end{equation*}

There are a total of 40 fit parameters. Each structural component in the nearest neighbor model has two parameters, enthalpy and entropy. The model has 10 nearest neighbor terms for stacks of Watson–Crick pairs (20 parameters), terminal AU pairs (2 parameters), terms for intermolecular initiation (2 parameters) and eight lengths of disordered internal loops (16 parameters). Fitting was done with the trust-region algorithm (42) as implemented in the Intel MKL library (43).

### Two-state fitting procedure

The two-state fitting procedure of Xia *et al*. was used on the set of 34 available optical melting experiments (16). Enthalpy, entropy, and free energy data and their corresponding errors for all 34 sequences were collected from the original publications (Table 1). Then these experimental quantities were written as a sum of contributions of individual nearest neighbor parameters depending on the sequence. The values of nearest neighbor parameters were then determined from an error-weighted linear least-squared fit, performed with the DGELSS routine from the LAPACK library (44). The errors in this method were estimated using the same approach as in the original work, i.e. as the square-root of the diagonal elements of the variance–covariance matrix produced from DGELSS routine.

### Statistical tests

To test the statistical significance of improved accuracy with the new method, a one-tailed, paired *t*-test with type I error rate set to 0.05 was performed. When comparing the partition-function fits to the literature parameters, the null hypothesis is that the accuracy of predictions with the new parameters are not improved compared to the predictions made with the literature parameters. If the calculated *P* value is <0.05 then the null hypothesis is rejected and it is concluded the performance of the partition-function-fit parameters is significantly better. The *P* values were calculated with Microsoft Excel 2010.

### Testing the quality of fit by jackknife

To test the quality of partition function fit and estimate the uncertainties of fitted parameters, jackknife resampling was performed (45). In the jackknife method, the fitting is performed on all but one duplex and then the resulting parameters are used to test the performance on the duplex that was left out of the fitting. This is then repeated for all duplexes. This method allows testing of performance of the new fit procedure on each duplex without introducing the bias from using the data for both fitting and testing. The uncertainty of the fitted parameters is taken as the standard deviation of the fitted parameters from jackknife resampling.

## RESULTS

### Fitting

Optical melting data (16,35,36) for 34 unique duplexes and 285 individual melts (Table 1) were used for fitting the nearest neighbor parameters. All data were collected in a standard buffer system with 1 M Na^+^ and pH 7. In all melts, the original absorbance versus temperature curves were transformed into fraction of base pairing as a function of temperature using the procedure described in the Materials and Methods.

Nearest neighbor parameters were fit to the optical melting data using a non-linear least-squares method, as detailed in the Materials and Methods. The fitting parameters are enthalpy and entropy changes of Watson–Crick pair stacks, initiation, terminal AU, and disordered loops, for a total of 40 parameters. Non-linear least-squares fitting requires initial estimates for the fitting parameters and does not guarantee the global minimum will be determined. The two-state-derived values of nearest neighbors (16) were used as starting values. Additionally, to test convergence, another 100 fits were performed starting with the two-state nearest neighbor parameters randomly perturbed with a uniform variate by up to 20% of their initial value. This procedure produces high initial values for the square root of squared residuals, at least 3 times higher than the two-state derived parameters in 90 of 100 cases. Remarkably, the final square root of the sum of squared residuals in all runs were the same to within 0.001, and the values of fitted free energies of Watson–Crick, terminal AU and initiation parameters at 37°C were within 0.01 kcal/mol (lower than the error reported for the 2-state-derived parameters) in all 100 fits. In contrast, fitted values for the disordered loop parameters vary widely. Their standard deviations generally have large magnitude (Table S2B in Supplementary Data). The quality of fit, however, as quantified by the square root of the sum of squared residuals, is similar across the 100 fits although these disordered loop parameter values vary widely. This suggests that these parameters have little effect on the stability of duplexes, because there is a low probability of forming structures with internal loops in the fitting dataset composed of short duplexes with only five to ten Watson–Crick base pairs. These results indicate that the fitting procedure produces values that are converged. The results of multiple starting point fits are presented in the Supplementary Data in Tables S2A and S2B.

The literature set of nearest neighbor parameters was derived from a database of 90 unique duplexes, analyzed by two-state fitting (16). Expansion of the database by 22 duplexes had negligible effect on the parameters (17). Here, however, the original melting data (UV absorbance as a function of temperature) for only 34 unique duplexes was available. To facilitate comparison of the two fitting methods, a nearest neighbor parameter set was also derived using the same procedure as in the previous work (16), but using only the 34 duplexes available for the partition function-based fits. As shown in Table 2, the fit performed with the subset of duplexes results in small deviations from the literature parameters.

**Table 2.** Comparison of the free energy changes at 37°C of the literature nearest neighbor (NN) parameters derived with the two-state assumption (16), nearest neighbor parameters derived using the same procedure, but with the data from 34 duplexes available in this work, and the nearest neighbor parameters derived by fitting to melting data using the partition function. Error estimates for the parameters from the partition function are from jackknife estimates. The error estimates for the 34-duplex two-state fit are larger in magnitude than for the literature two state parameters because error estimates are standard errors of the mean, which reduce in magnitude when more independent observations are available. The second column gives the number of occurrences of each parameter in the set of 34 duplexes that were used in fitting. Detailed instructions and examples for using these parameters to predict helical stability are available on the Nearest Neighbor Database (NNDB) website, http://rna.urmc.rochester.edu/NNDB (1).

Parameter	Number of NN parameters in 34 fitted sequences	Δ*G*° (37°C) (kcal/mol)		
		NN two-state (90 duplexes)	NN two-state (34 duplexes)	NN Partition (34 duplexes)
Initiation	34	4.1 ± 0.2	3.3 ± 0.5	3.6 ± 0.1
AA/UU	19	−0.93 ± 0.03	−1.08 ± 0.08	−1.15 ± 0.02
AU/UA	13	−1.10 ± 0.08	−1.2 ± 0.2	−1.20 ± 0.04
UA/AU	15	−1.33 ± 0.09	−1.3 ± 0.2	−1.30 ± 0.04
CU/GA	33	−2.08 ± 0.06	−1.8 ± 0.3	−1.82 ± 0.03
CA/GU	39	−2.11 ± 0.07	−1.8 ± 0.3	−1.97 ± 0.03
GU/CA	31	−2.24 ± 0.06	−2.3 ± 0.4	−2.25 ± 0.05
GA/CU	38	−2.35 ± 0.06	−2.4 ± 0.4	−2.52 ± 0.05
CG/GC	10	−2.36 ± 0.09	−2.0 ± 0.6	−2.25 ± 0.07
GG/CC	9	−3.26 ± 0.07	−3.4 ± 0.1	−3.68 ± 0.05
GC/CG	11	−3.42 ± 0.08	−3.5 ± 0.7	−3.7 ± 0.1
Terminal AU	47	0.45 ± 0.04	0.57 ± 0.07	0.59 ± 0.02

The square root of the sum of squared residuals of the fraction of bases paired, the quantity that was used to measure the quality of fit, decreased from 15.605 when using the literature two-state parameters (16) to 9.594 with the partition function-fitted parameters, an improvement of 38.5%. The square root of the sum of squared residuals using the two-state fitted parameters with the set of 34 duplexes and using the partition function to estimate the melting behavior was 14.348. Table 2 gives the values of free energy changes at 37°C of the literature nearest neighbor parameters and those fitted using the partition function and two-state models, applied to only the 34 available duplexes. Reported uncertainties are standard deviations of the parameters from the jackknife procedure for the partition function fit.

The quantities fit were temperature-independent enthalpy and entropy changes, but they are highly correlated because the melting temperatures of the duplexes in the dataset are all in a relatively narrow range (16). Because of this correlation, the values of enthalpy and entropy changes are not meaningful when used independently of each other. Calculating the free energy change at a specific temperature using $\Delta {G^\circ}(T) = \Delta {H^\circ} - T\Delta{S^\circ}$ removes the correlation. Additionally, the free energy changes are predicted with higher accuracy as compared to enthalpy changes and entropy changes (16).

The parameters for internal disordered loops are given in Table S2B in the Supplementary Data. These terms do not have an equivalent parameter in the two-state-derived parameters, which instead includes parameters for structured, sequence-specific loops (1,18,20). Furthermore, based on the multiple starting point fits, the disordered loop parameters do not appear to have significant effect on the results of the fit.

### Benchmarks of fitted parameters

**Figure 1.**
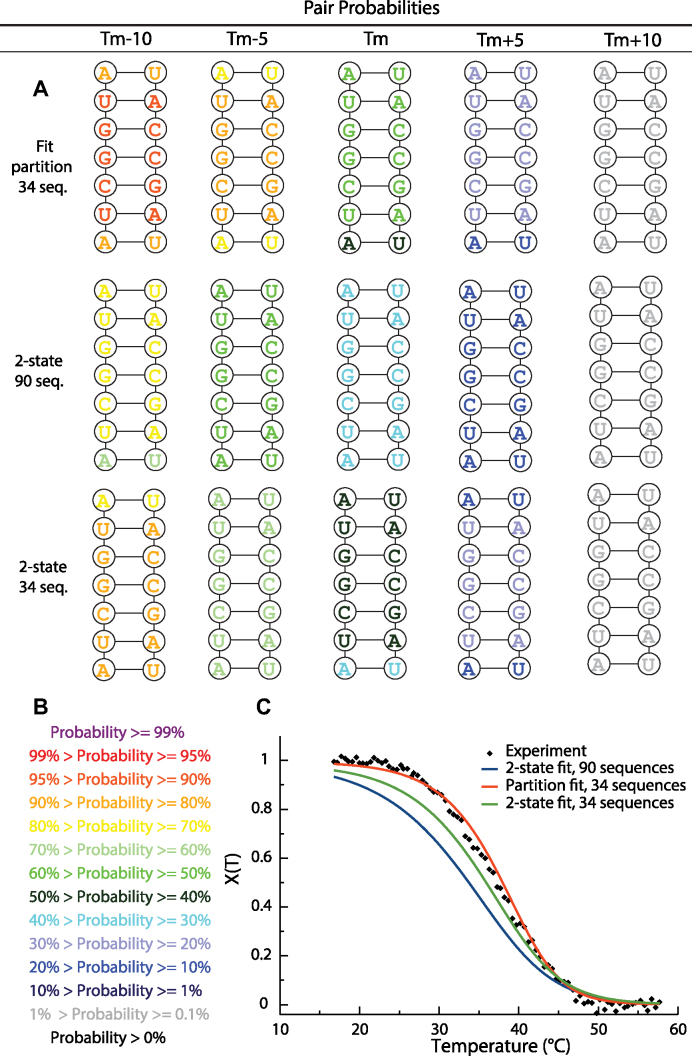
Comparison of melting for duplex 5′-AUCGGUA/3′-UAGCCAU with total strand concentration of 6 μM. Panel **A** provides pair probabilities at the experimental melting temperature (*T_m_* = 37.1°C), *T*_m_ ±5°C and *Tm* ±10°C using the literature two-state-derived parameters (16) and the newly fitted parameters. Panel **B** is the pair probability color annotation key. In panel **C**, the estimated melting curves are compared to the fraction of maximal base pairs as a function of temperature derived from the optical melting curves (see Materials and Methods). Diamonds are the experimental melting data. The red line is the prediction using the newly fitted parameters, the blue line is the prediction using the literature two-state-derived nearest neighbor parameters (16) and the green line is the prediction using the parameters from the two-state fit on 34 duplexes available in this work. All melting curves were computed using a partition function. Melting temperatures for the experiment, the partition function parameters, literature two-state parameters, and two-state parameters with 34 duplexes are 37.1, 38.0, 33.7 and 35.9°C, respectively. RMSDs between experimental and melting curves from two-state fit to 90 duplexes (blue line), partition function fit to 34 duplexes (red line) and two-state fit to 34 duplexes (green line) were 0.12, 0.03 and 0.06 respectively.

Figure 1 and Supplementary Figure S4 show the results for duplex 5′-AUCGGUA/3′-UAGCCAU, comparing the new method to the two-state fits. Panel B of Figure 1 compares the experimental melting curve for the duplex at 6 μM strand concentration along with melting curves predicted with two-state parameters from 90 duplexes (16) or from 34 duplexes and the new parameters fit with the partition function approach. The experimentally-determined melting temperature was 37.1°C, and the melting temperatures determined with the literature two-state-derived parameters, new parameters fit using the partition function and two-state parameters fit using 34 duplexes were 33.7, 38.0 and 35.9°C, respectively. In panel A of Figure 1, pair probabilities were estimated at the experimental melting temperature (37.1°C) and at ± 5 and ±10°C from that temperature. In Supplementary Figure S4, the pairing probabilities were estimated for temperatures from 15 to 60°C. Figure 1 and Supplementary Figure S4 illustrate that the newly fitted parameters describe melting behavior more accurately than the prior two-state-derived parameters. In addition, all parameter sets predict terminal base pairs have lower base pairing probabilities than pairs interior to the helix, which is a result of the expected end fraying of the helix.

To test the performance of the newly fitted parameters, their predictions were compared with predictions of literature parameters (16) derived using the two-state model and 90 duplexes or the two-state fit using the 34 duplexes available now. Because all 34 duplexes were used for deriving the parameters, the predictions for the two sets of parameters derived here (the partition function fit and the fit using the two-state approximation for the 34 duplexes) were made using the jackknife method. In jackknife, predictions for each duplex are made by fitting to all but that one duplex and then testing on that duplex. In this way, training and testing are not done with the same sequences. The average of the jackknife parameters is given in Supplementary Tables S2A and S2B for enthalpies, entropies and free energies. As expected, the average parameter values for pair stacks across the jackknife calculations match closely (ΔΔ*G*°_37_ < 0.1 kcal/mol) the parameters from the fit to all duplexes.

The partition function derived in this work was used to make predictions with all three sets of nearest neighbor parameters. Nearest neighbor parameters for the 2 two-state models do not have loop parameters that are used in the partition function. Furthermore, two-state models predict that no loops should form during the folding/unfolding process. Therefore, we set the enthalpy and entropy values for all loop neighbor parameters for the two-state models to 10.0 kcal/mol and −1.0 e.u., respectively, producing an approximately 10 kcal/mol free energy change in the range of temperatures used. This high value prevents loop formation in these two models that do not allow loop formation.

**Figure 2.**
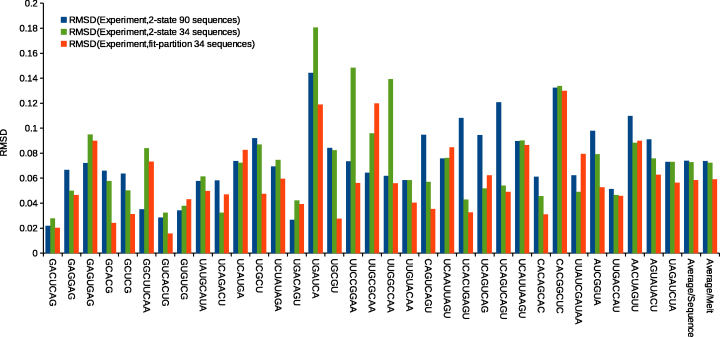
Comparison of root mean square deviations (RMSD) of fraction of bases paired between experimental and estimated optical melting curves. Three sets of parameters were compared: the literature two-state parameters (blue bars), the parameters from two-state fit performed with 34 duplexes (green) and the fit with the partition function (red). The predictions of two sets of parameters derived here (green and red bars) were derived using the jackknife method. The final two columns are comparisons of the averages per unique duplex and averages per melt, as each duplex had multiple melts. Note that the RMSD is calculated for fraction of bases paired, which is bounded between 0 and 1, so the improvement in the fits is substantial. Sequences are written with 5′ end at top.

Figure 2 compares the root-mean-square deviation (RMSD) between experimental data and predictions for all three models averaged across all melts for each unique duplex. The two last clusters of bars plotted in Figure 2 are the average over the melts belonging to the 34 unique duplexes and the average over all 285 optical melting curves. Relative to the literature parameters (16), the set of parameters derived using the partition function produces better agreement with the experimental data for 26 of 34 sequences used in fitting and for both averages. Parameters derived using the two-state model and 34 duplexes also perform better than the literature set (16) on average, but here the difference is smaller. A pairwise t-test indicates that the difference in performance between literature and partition function-fitted parameters is statistically significant (*P*<0.05), while the difference in performance between the literature two-state and two-state derived using the 34 duplexes is not statistically significant. When the t-test was performed on individual melts of each duplex, of the eight duplexes that performed worse with the partition function-derived parameters, five were statistically significant (5′-GAGUGAG-3′, 5′-GGCUUCAA-3′, 5′-UGACAGU-3′, 5′-UUGCGCAA-3′ and 5′-UUAUCGAUAA-3′). Thus, the new parameters improve prediction of the melting curves used for fitting and testing.

**Figure 3.**
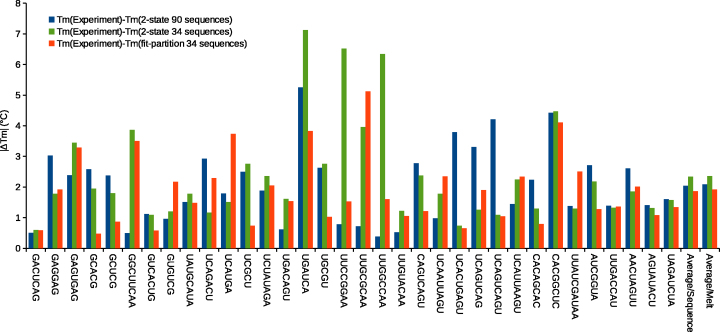
Comparison of absolute values of differences between predicted melting temperatures and measured melting temperatures. Blue bars are the differences between melting temperatures calculated directly from melting experiments and predicted using the literature two-state model derived nearest neighbor parameters (16). Green bars are the differences between experimental melting temperatures and melting temperatures predicted using the two-state model and the 34 duplexes used in this work. Red bars are differences between experimental melting temperatures and melting temperatures calculated using the parameters derived in this work by fitting to the partition function. The predictions of two sets of parameters derived here (green and red bars) were derived using the jackknife method. The last two bars are averages over the 34 unique duplexes and over all individual melts, as each duplex had multiple melts. Sequences are written with 5′ end at top.

Another test for performance of the newly fitted parameters is prediction of melting temperatures. Melting temperatures were calculated directly from the melting curves by linear interpolation (described in the Materials and Methods) to the temperature value where the fraction of strands in double helix is 0.5. Figure 3 presents the mean absolute value of the difference between melting temperature calculated directly from the experimental melting curves and melting temperatures estimated with the three models. Mean values calculated without taking the absolute value of differences are given in Figure S5 in the Supplementary Data. As in Figure 2, results for the partition function and two-state fits are obtained from jackknife resampling where the fit was done for all but one duplex and then the parameters were used to predict melting temperature of the melts of the duplex that was left out. Parameters from the fit using the partition function outperform the predictions of the literature two-state models for 20 of 34 duplexes and for averages over duplexes or over melts. Unlike the RMSD tests in Figure 2, literature two-state parameters outperform the two-state fit of 34 duplexes. The pairwise t-tests indicate that the improvement of the partition-function-fit parameters is statistically significant compared to the two-state fit to 34 duplexes, but not compared to the literature parameters. Of the 14 duplexes where the partition function performs worse than the literature two-state parameters, the differences are statistically significant in 10 duplexes (5′-GAGUGAG-3′, 5′-GGCUUCAA-3′, 5′-UGACAGU-3′, 5′-UUGCGCAA-3′, 5′-UUAUCGAUAA-3′, 5′-GUGUCG-3′, 5′-UCAUGA-3′, 5′-UUCCGGAA-3′, 5′-UUGGCCAA-3′ and 5′-UCAAUUAGU-3′). Thus, newly derived parameters were better able to describe melting temperatures, although the improvement is not statistically significant compared to the literature set (16).

Figure S6 in Supplementary Data also shows that using the partition function instead of two-state model even with the original two state parameters (16) can predict melting curves and melting temperatures more accurately.

## DISCUSSION

A novel method is introduced for deriving nearest neighbor parameters for nucleic acids folding stability by using a partition function model to directly fit the parameters to optical melting data. This procedure avoids the two-state assumption. It also resolves the intrinsic inconsistency in using parameters fit to a two-state model when using partition functions to predict folding including partially unfolded states.

Prior optical melting studies on duplexes compared the agreement between enthalpy changes determined from the mean of the fits to individual curves and the fits to van’t Hoff plots (16,23). When the fits disagreed by over 15%, it was concluded the melting for that sequence was not two state. That condition (agreement within 15% in enthalpy change) was necessary but not sufficient to conclude that melting was two state (23). In light of results here, many of the duplexes that were assumed to melt with two-state behavior were melting with some degree of end fraying.

Free energy values at 37°C of the literature two-state parameters from 90 duplexes (16), new parameters fit from 34 duplexes with a partition function, and two-state parameters fit from 34 duplexes are given in Table 2. The largest differences between literature (16) and partition function values are 0.5 and 0.4 kcal/mol in initiation and GG/CC stacking parameters, respectively. For five other parameters, the difference is 0.1 kcal/mol or less. The direction of change is not uniform; four of the fitted parameters are higher than the prior, eight are lower, and on average the newly fitted parameters have 0.09 kcal/mol lower magnitudes. These changes suggest a subtle rearrangement of relative interaction energies that results in a better overall description of melting behavior. It is also worth noting that CG/GC is still less stable than either GG/CC or GC/CG. It is this prominent difference that requires the use of nearest neighbor stacks to estimate helix stability as opposed to a simpler base pair composition model.

Parameters derived using the two-state assumption, with 34 duplexes, differ from the literature set derived using the two-state assumption with 90 duplexes. The direction of change generally follows the fit using the partition function, indicating perhaps a bias in some nearest neighbor parameters caused by the different relative abundance of parameters in the 34-duplex dataset compared to the 90-duplex dataset.

Uncertainties for the partition function-fit parameters are approximately the same magnitude as the two state-parameters derived using the original 90 duplex dataset, although the uncertainties of the two-state parameters from the 34 duplex fit are larger by two times or more in magnitude (Table 2). These larger uncertainties are a result of the smaller dataset used in fitting (34 vs 90 duplexes). In addition, both parameter sets derived using the two-state approximation include uncertainties from both experimental data (from van’t Hoff plots) and from the linear least squares fit. Errors presented in Table 2 for these two methods include both contributions, as the data used in fitting was weighted by uncertainties (16). On the other hand, the partition function fit was performed directly on melting curves and the uncertainty in measuring absorbance contributes negligibly to prediction of free energies, less than 0.005 kcal/mol according to Xia *et al*. (16). The non-linear least-squares fit used to determine the partition function-fit parameters does not allow accurate determination of uncertainties especially when the fitted parameters are correlated such as enthalpy and entropy changes (16). Therefore, the standard deviations of parameter estimates from jackknife resampling were used to estimate the uncertainties. These estimates (≤0.1 kcal/mol) account for uncertainty from random experimental errors and from systematic errors due to neglect of non-nearest neighbor effects.

Relative to the partition-function-fit parameters, five duplexes more closely fit the melt (as measured by RMSD) with significance and ten of the 34 duplexes had better predicted melting temperature with significance using the literature two-state parameters (16). This difference could be caused by imperfect fitting to the lower and upper baselines of the melting curves. In these duplexes, the parameters from the two-state fit to the 34 duplex set also perform worse than parameters from the fit to the partition function. Therefore it is possible that the cause of the worse predictions is not enough data in the 34 duplex set to model certain nearest neighbor parameters correctly when fitting by linear regression to two-state-derived curve fits. Note that this is less of an issue for the partition function fit, where the parameters are fit to optical melting data directly and hence there are many more points of data (39,382) in the fit. As Figures 2 and 3 show, parameters from the fit to the partition function outperform the two-state parameters derived using the same dataset, indicating that the partition function is better able to describe the behavior of even these simple duplexes.

The robustness of fit was tested by performing jackknife resampling for both the partition function fit and the two-state fit on the available 34 duplex dataset. By performing the fit on all but one duplex and then testing the parameters on that duplex, an independent estimate of the performance of the model on all duplexes can be determined. Overall, parameters from the partition function fit better describe both melting curves and melting temperatures than either of the two-state models. Including data from more duplexes and getting a more equal distribution of nearest neighbor parameters in the database would likely further improve the accuracy of predictions.

In this work, and in the conventional two-state fits to optical melting data, the heat capacity change, *ΔCp*, is assumed to be zero, i.e. the Δ*H*° and Δ*S*° are assumed to be temperature independent. A non-zero heat capacity change can be found by performing optical melting as a function of strand concentration for duplexes (19,46,47) or by performing isothermal titration calorimetry as a function of temperature (48,49). On average, the heat capacity change for duplexes as determined from optical melting in 1 M NaCl is −365 ± 64 cal K^−1^ mol^−1^ (21,47). It is possible that this heat capacity change is a result of base stacking in the unpaired strands or from interactions of duplexes with the solvent (47,50–55). The effects of the heat capacity change on enthalpy and entropy, however, are opposite in their impact on free energy change; i.e. they compensate each other because the enthalpy change and entropy change both decrease as temperature is increased (51). Thus, the parameters fit to the partition function are most accurate around the melting temperatures of the duplexes, but are probably reasonable in the temperature range of 10°C to 60°C where the effect of heat capacity change on free energy change is <0.5 kcal/mol (21). A future extension of the partition function fitting approach could be to include a fit of sequence-dependent terms for heat capacity change, but that would require a larger optical melting dataset than is currently available.

This work focused on RNA Watson–Crick duplexes, but subsequent studies could fit the full nearest neighbor model including terms for GU pairs and loops. Given the improved estimates of melting temperatures and melting curve shape, it is also expected that the revised parameters would improve the accuracy of RNA secondary structure prediction. That hypothesis can only be tested once a full set of loop parameters are also derived without assuming two-state melting. At the current time, these new Watson–Crick parameters should not be used in conjunction with the larger set of nearest neighbor parameters for loops because the loop parameters were fit with a dependency on the Watson–Crick parameters. These fits could also be tailored by learning parameter values that also result in improved RNA secondary structure prediction (56). Additional future work should fit the DNA nearest neighbor parameters without the two state assumption.

## Supplementary Material

Supplementary Data
