# Supplementary material for: Improving RNA nearest neighbor parameters for helices by going beyond the two-state model
Source: Nucleic Acids Res. 2018 May 1;46(10):4883–92. doi: 10.1093/nar/gky270 (PMC6007268; doi:10.1093/nar/gky270)
Supplement: Supplementary Data [file gky270_supp.pdf]

Supplementary Data to accompany:

Improving RNA Nearest Neighbor Parameters for Helices by Going Beyond the Two-State Model

Aleksandar Spasic, Kyle D. Berger, Jonathan L. Chen, Matthew G. Seetin, Douglas H. Turner, David H. Mathews

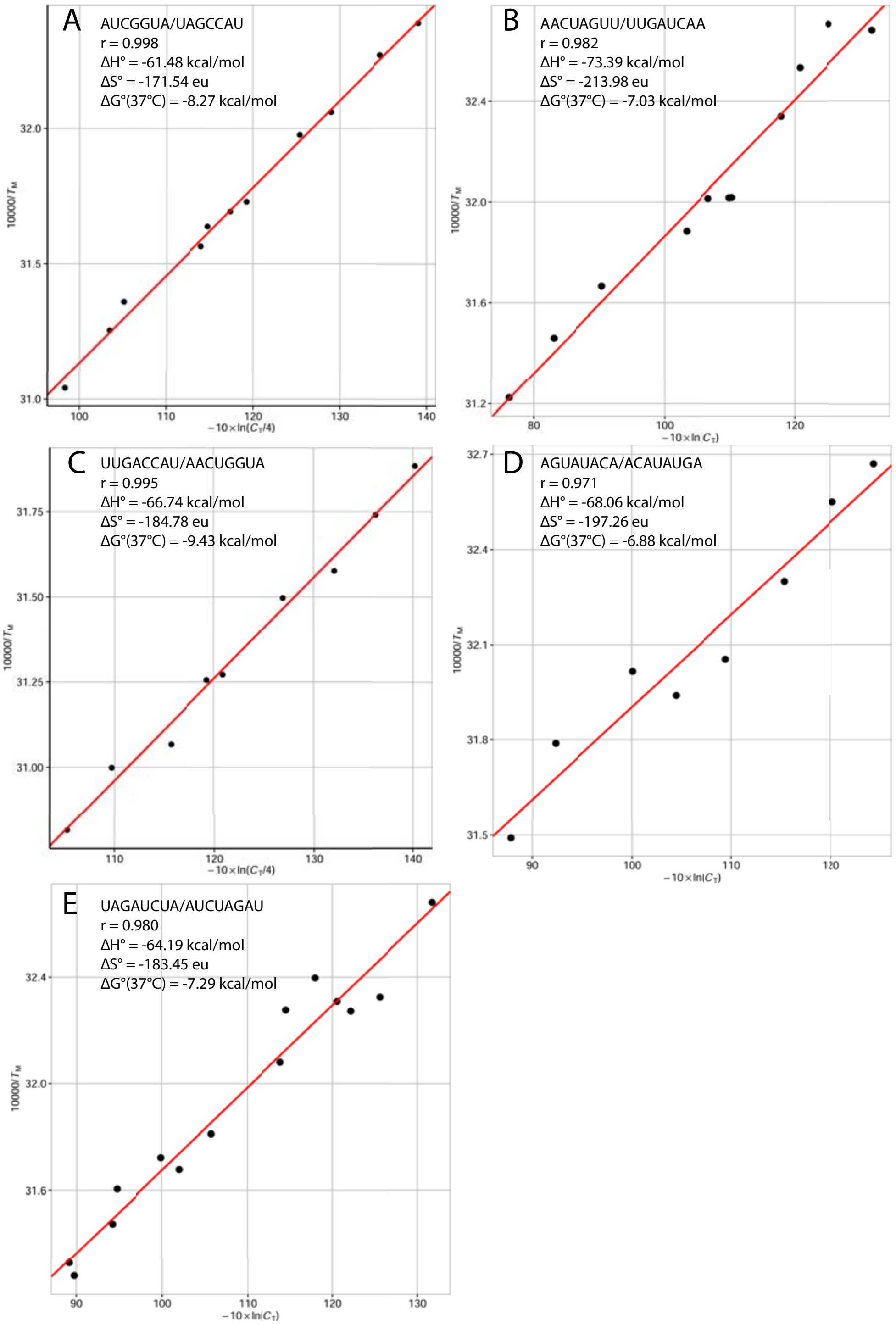

Figure S1. Plots of logarithm of strand concentration ($\ln(C_t)$) as a function of inverse of melting temperature ($1/Tm$), which were used to determine the enthalpy change and entropy change using the two-state fit for the five sequences melted in this work. Panel A is AUCGGUA, panel B is AACUAGUU, panel C is UUGACCAU, panel D is AGUAUACA and panel E is UAGAUCUA melting data. Corresponding R^2 values and thermodynamic parameters are given in each plot and Table S1. All melting curves were measured at 260 nm.

Table S1. Free energy change at 37 °C, enthalpy change, and entropy change derived from the two-state fit for the five sequences melted in this work.

Sequence	ΔG° (37 °C) (kcal/mol)	ΔH° (kcal/mol)	ΔS° (e.u.)	Tm (°C) at 0.1 mM total strand concentration
AUCGGUA	-8.27±0.03	-61.48±1.401	-171.54±4.445	46.0
UUGACCAU	-9.43±0.08	-66.76±2.495	-184.78±7.821	51.1
AACUAGUU	-7.03±0.09	-73.39±4.663	-213.98±14.94	42.8
AGUAUACU	-6.88±0.165	-68.06±6.825	-197.26±21.87	42.6
UAGAUCUA	-7.29±0.09	-64.19±3.782	-183.45±12.07	45.0

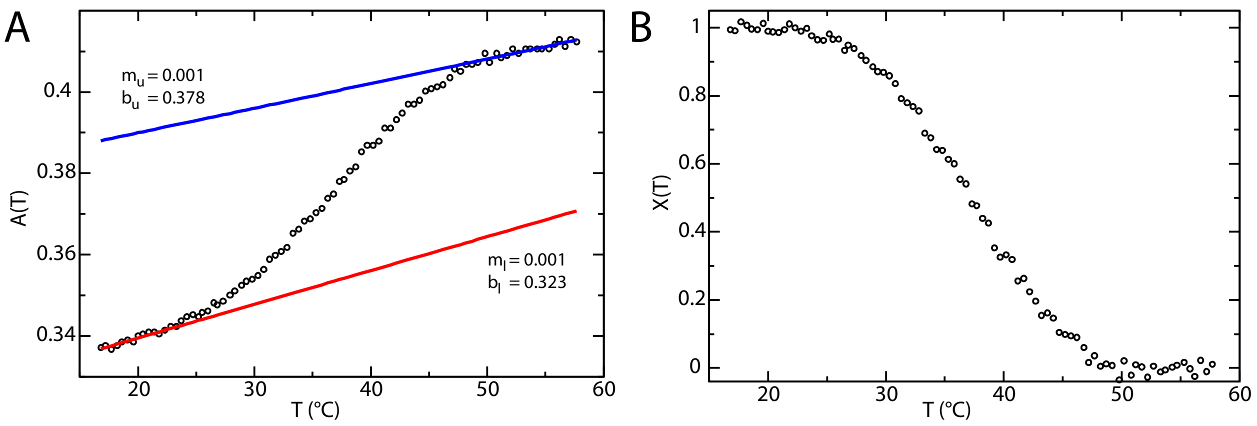

Figure S2. The optical melting data for duplex 5'-AUCGGUA/3'-UAGCCAU with total strand concentration of 6 μM. (A) The measured UV absorbance at 260 nm as a function of temperature (circles). Also shown are the fit lower baseline (red) and upper baseline (blue). (B) Using the melting data and the baselines, the fraction of maximal pairs formed at temperature *T*, $X_{melt}(T)$, was calculated.

Calculating Hypochromicity. Percent hypochromicity of a sequence was calculated (1) as,

$$\%\ Hypocromicity = (100\ \%)\frac{A(melted\ species) - A(double\ strand)}{A(double\ strand)}$$

where $A(melted\ species)$ and $A(double\ strand)$ were absobances of melted species (taken here to be an absorbance at 90 °C) and absorbance of double helix (taken here to be absorbance at 10 °C). Linear regression to the ratio of AU to GC pairs produces a fit with a coefficient of determination, R^2, = 0.09 and F observed value of 28.46. The p-value of the F test was 1.96×10^{-7}. Given the small magnitude of the correlation, the relative hypochromicities of AU and GC base pairs could not be reliably determined from sequence. A plot of hypochromicity as a function of AU/GC ratio is in Figure S2.

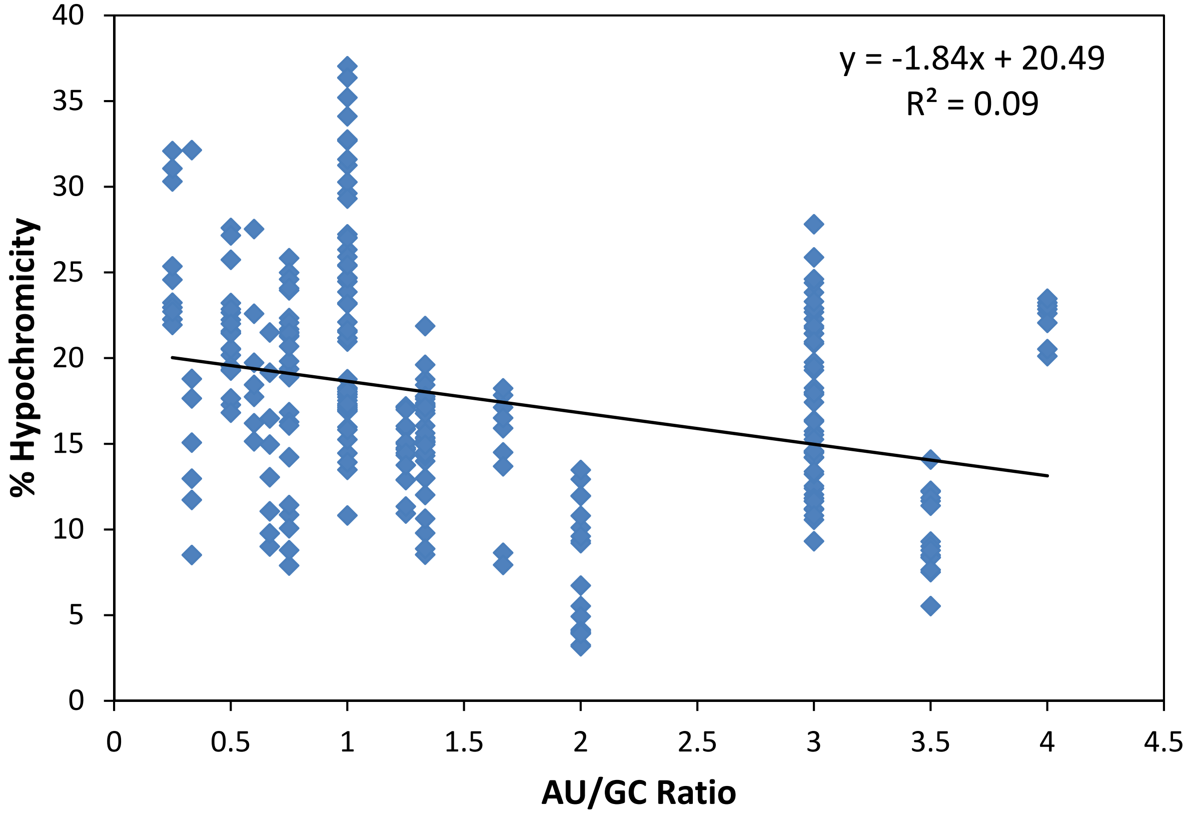

Figure S3. Plot of % hypochromicity of all 285 optical melting curves used in this work as a function of their ratio of AU to GC base pairs.

Calculation of Partition Function by Recursion. The partition function is calculated from $Q_{L,i}$ and $Q_{R,i}$, which are partial partition functions for all structures with a base pair at position i and that are accumulated from the left (5' direction of top strand) and from the right (3' direction for the top strand), respectively. These terms are calculated recursively, starting from the 5' or 3' ends of sequence using the following equations:

$$Q_L(i) = Q_L(i-1)K_{stack}(i) + K_{init}K_{AUend(if\ i=AU)} + \sum_{1\le k<i-1} Q_L(k)K_{loop}(k,i)K_{AUend(if\ i=AU)}K_{AUend(if\ k=AU)}$$

where i and k are indices for base pairing position where the 5' end of the top strand is position 1. The first term is the stack of the ith pair on the previous, second is for the helix starting at the ith position and the last term is for an internal loop closed by pairs at position i and k. $K_{loop}(k,i)$ is the equilibrium constant for an internal loop closed by pairs at the k and i position, $K_{stack}(i)$ is the equilibrium constant for the ith pair stacked on the pair at the $i-1$ position, K_{init} is the equilibrium constant for initiation and K_{AUend} is the equilibrium constants for a terminal AU base pair. $Q_L(0)$ is initialized as zero.

Similarly:

$$Q_R(i) = Q_R(i+1)K_{stack}(i+1) + K_{AU(if\ i=AU)} + \sum_{i+1\le k<L} Q_R(i,k)K_{loop}K_{AUend(if\ i=AU)}\,K_{AUend(if\ k=AU)}$$

$Q_R(L+1)$ is initialized as zero, where L is the length of the helix. The equilibrium constants are calculated from free energy changes using the familiar relationship between equilibrium constant, free energy and enthalpies and entropies:

$$K_l = \exp\left(\frac{-\Delta G_l^0(T)}{RT}\right) = \exp(\frac{-\Delta H_l^0(T)}{RT} + \frac{\Delta S_l^0(T)}{R})$$

where l denotes the motif. The total partition function is calculated by summing:

$$Q = 1 + \sum_{1\le k\le L} Q_L(k)K_{AUend(if\ k=AU)}$$

$Q_L(i)$ and $Q_R(i)$ are defined such that the product, $Q_L(i)Q_R(i)$ is the partition functions over all structures with a base pair at the ith position. Hence, $Q_L(i)Q_R(i)/Q$ is the probability of base pairing at the ith position, given that at least one base pair exists.

Table S2A. Comparison of enthalpies and entropies obtained from the 100 fits starting from the literature two-state parameters perturbed by up to 20% and parameters derived from the jackknife procedure. Two-state-derived parameters for disordered loops do not exist.

Parameter	Two-state-derived parameters (kcal/mol for ΔH° and e.u. for ΔS°)	(Fit starting from two-state parameters) ± (standard deviation of jackknife procedure) (kcal/mol for ΔH° and e.u. for ΔS°)	(Average of jackknife procedure) ± (standard deviation of jackknife procedure) (kcal/mol for ΔH° and e.u. for ΔS°)	(Average from 100 fits starting from perturbed parameters) ± (standard deviation) (kcal/mol for ΔH° and e.u. for ΔS°)
Initiation ΔH°	3.61	9 ± 3	9 ± 3	9 ± 3
Initiation ΔS°	-1.50	17 ± 8	18 ± 8	18 ± 9
AA/UU ΔH°	-6.82	-9.8 ± 0.5	-9.8 ± 0.5	-9.8 ± 0.1
AA/UU ΔS°	-19.00	-28 ± 2	-28 ± 2	-27.8 ± 0.3
AU/UA ΔH°	-9.38	-23 ± 2	-23 ± 2	-23.1 ± 0.3
AU/UA ΔS°	-26.70	-71 ± 5	-70 ± 5	-71 ± 1
UA/AU ΔH°	-7.69	-7.8 ± 0.8	-7.9 ± 0.8	-8 ± 1
UA/AU ΔS°	-20.50	-21 ± 3	-21 ± 3	-21 ± 3
CU/GA ΔH°	-10.48	-16.3 ± 0.5	-16.3 ± 0.5	-16.3 ± 0.2
CU/GA ΔS°	-27.10	-47 ± 2	-47 ± 2	-46.7 ± 0.8
CA/GU ΔH°	-10.44	-12.1 ± 0.7	-12.2 ± 0.7	-12.1 ± 0.3
CA/GU ΔS°	-26.90	-33 ± 2	-33 ± 2	-32.6 ± 0.9
GU/CA ΔH°	-11.40	-14 ± 1	-14 ± 1	-14.4 ± 0.7
GU/CA ΔS°	-29.50	-39 ± 4	-38 ± 4	-39 ± 2
GA/CU ΔH°	-12.44	-11.6 ± 0.9	-11.6 ± 0.9	-12 ± 1
GA/CU ΔS°	-32.50	-29 ± 3	-29 ± 3	-30 ± 4
CG/GC ΔH°	-10.64	-19 ± 1	-19 ± 1	- 19.2± 0.1
CG/GC ΔS°	-26.70	-55 ± 5	-56 ± 5	-54.8 ± 0.3
GG/CC ΔH°	-13.39	-21 ± 1	-21 ± 1	-21.0 ± 0.4
GG/CC ΔS°	-32.70	-56 ± 4	-55 ± 4	-56 ± 1
GC/CG ΔH°	-14.88	-16 ± 2	-16 ± 2	-16 ± 1
GC/CG ΔS°	-36.90	-40 ± 5	-40 ± 5	-41 ± 5
Terminal AU ΔH°	3.72	3.1 ± 0.4	3.1 ± 0.4	3.12 ± 0.02
Terminal AU ΔS°	10.50	8 ± 1	8 ± 1	8.16 ± 0.06
1×1_loop ΔH°	N/A	15 ± 3	2 ± 3	1 ± 5
1×1_loop ΔS°	N/A	-21 ± 2	-19 ± 2	-16 ± 20
2×2_loop ΔH°	N/A	-2 ± 3	-4 ± 3	-3 ± 21
2×2_loop ΔS°	N/A	-37 ± 1	-38 ± 1	-35 ± 18
3×3_loop ΔH°	N/A	-4 ± 4	5 ± 4	6 ± 13
3×3_loop ΔS°	N/A	-11 ± 5	-11 ± 5	-7 ± 7
4×4_loop ΔH°	N/A	2 ± 4	-2 ± 4	0 ± 44
4×4_loop ΔS°	N/A	-28 ± 2	-29 ± 2	-28 ± 16

5×5_loop ΔH°	N/A	-5 ± 3	-5 ± 3	-7 ± 7
5×5_loop ΔS°	N/A	-29 ± 1	-29 ± 1	-31 ± 17
6×6_loop ΔH°	N/A	6 ± 11	-2 ± 11	-2 ± 11
6×6_loop ΔS°	N/A	-10 ± 27	-16 ± 27	-16 ± 27
7×7_loop ΔH°	N/A	-2 ± 2	-2 ± 2	-1.3 ± 0.6
7×7_loop ΔS°	N/A	-11.1 ± 0.5	-10.7 ± 0.5	-11 ± 1
8×8_loop ΔH°	N/A	-1 ± 1	-1 ± 1	-1.3 ± 0.4
8×8_loop ΔS°	N/A	-10.6 ± 0.1	-10.6 ± 0.1	-11 ± 1

Table S2B. Comparison of free energies at 37 ˚C obtained from the 100 fits starting from the literature two-state parameters perturbed by up to 20% and the jackknife procedure. Two-state derived parameters for disordered loops do not exist.

Parameter	Two-state derived parameters (kcal/mol)	(Fit starting from two-state parameters) ± (standard deviation of jackknife procedure) (kcal/mol)	(Average of jackknife procedure) ± (standard deviation of jackknife procedure) (kcal/mol)	(Average from 100 fits starting from perturbed parameters) ± (standard deviation) (kcal/mol)
Initiation ΔG°	4.08	3.6 ± 0.1	3.6 ± 0.1	3.57 ± 0.09
AA/UU ΔG°	-0.93	-1.15 ± 0.02	-1.15 ± 0.02	-1.15 ± 0.01
AU/UA ΔG°	-1.10	-1.20 ± 0.04	-1.20 ± 0.04	-1.20 ± 0.01
UA/AU ΔG°	-1.33	-1.30 ± 0.04	-1.30 ± 0.04	-1.30 ± 0.01
CU/GA ΔG°	-2.07	-1.82 ± 0.03	-1.82 ± 0.03	-1.819 ± 0.003
CA/GU ΔG°	-2.10	-1.97 ± 0.03	-1.97 ± 0.03	-1.97 ± 0.01
GU/CA ΔG°	-2.25	-2.25 ± 0.05	-2.25 ± 0.05	-2.25 ± 0.03
GA/CU ΔG°	-2.36	-2.52 ± 0.05	-2.53 ± 0.05	-2.53 ± 0.03
CG/GC ΔG°	-2.36	-2.3 ± 0.1	-2.25 ± 0.07	-2.25 ± 0.01
GG/CC ΔG°	-3.25	-3.68 ± 0.05	-3.68 ± 0.05	-3.68 ± 0.01
GC/CG ΔG°	-3.44	-3.7 ± 0.1	-3.7 ± 0.1	-3.68 ± 0.05
Terminal AU ΔG°	0.46	0.59 ± 0.02	0.59 ± 0.02	0.589 ± 0.003
1×1_loop ΔG°	N/A	22 ± 4	8 ± 4	7 ± 4
2×2_loop ΔG°	N/A	9 ± 3	8 ± 3	8 ± 23
3×3_loop ΔG°	N/A	8 ± 4	9 ± 4	8 ± 14
4×4_loop ΔG°	N/A	11 ± 4	7 ± 4	9 ± 49
5×5_loop ΔG°	N/A	5 ± 3	4 ± 3	2 ± 3
6×6_loop ΔG°	N/A	9 ± 5	3 ± 5	3 ± 3
7×7_loop ΔG°	N/A	1 ± 2	2 ± 2	2.0 ± 0.8
8×8_loop ΔG°	N/A	2 ± 1	2 ± 1	2.0 ± 0.5

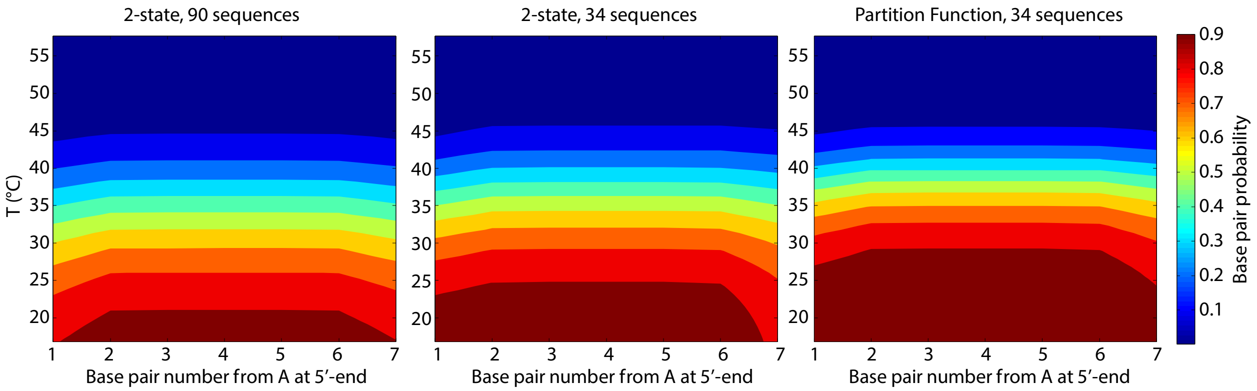

Figure S4. Plot of base pairing probability for duplex 5'-AUCGGUA/3'-UAGCCAU as a function of position in the helix and temperature for 6 µM total strand concentration. To estimate the pairing probabilities, a partition function calculation was performed for the literature two-state-derived nearest neighbor parameters (left), the parameters from the two-state fit on 34 duplexes available in this work (middle), and with the new fitted parameters (right). The experimental melting temperature of this duplex is 37.1°C. Substantially more end fraying is observed at low temperature with the parameter sets derived using the two-state assumption.

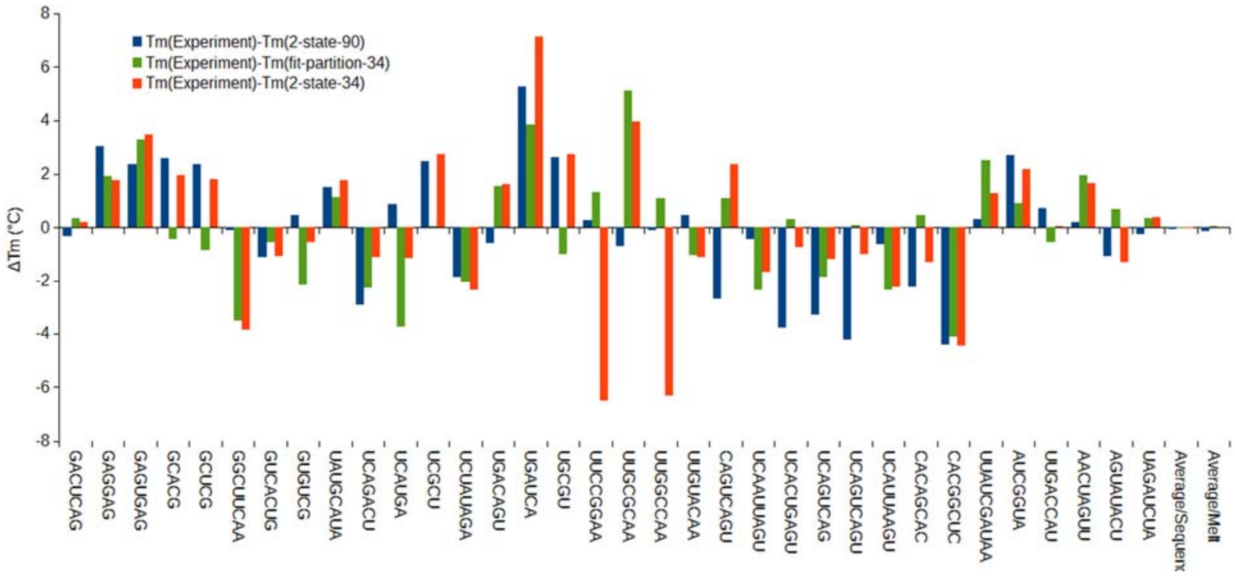

Figure S5. Comparison of differences between predicted melting temperatures and measured melting temperatures. Blue bars are the differences between melting temperatures calculated directly from melting experiments and predicted using the current two-state model-derived nearest neighbor parameters. Green bars are the differences between experimental melting temperatures and melting temperatures predicted using the two-state model and the 34 duplexes used in this work. Red bars are differences between experimental melting temperatures and melting temperatures calculated using the parameters derived in this work by fitting to the partition function. The predictions of two sets of parameters derived here (green and red bars) were derived using jackknife method. Data is given for all 34 sequences. The last two bars are averages over the 34 unique duplexes and over all individual melts, as each duplex had multiple melts.

A:

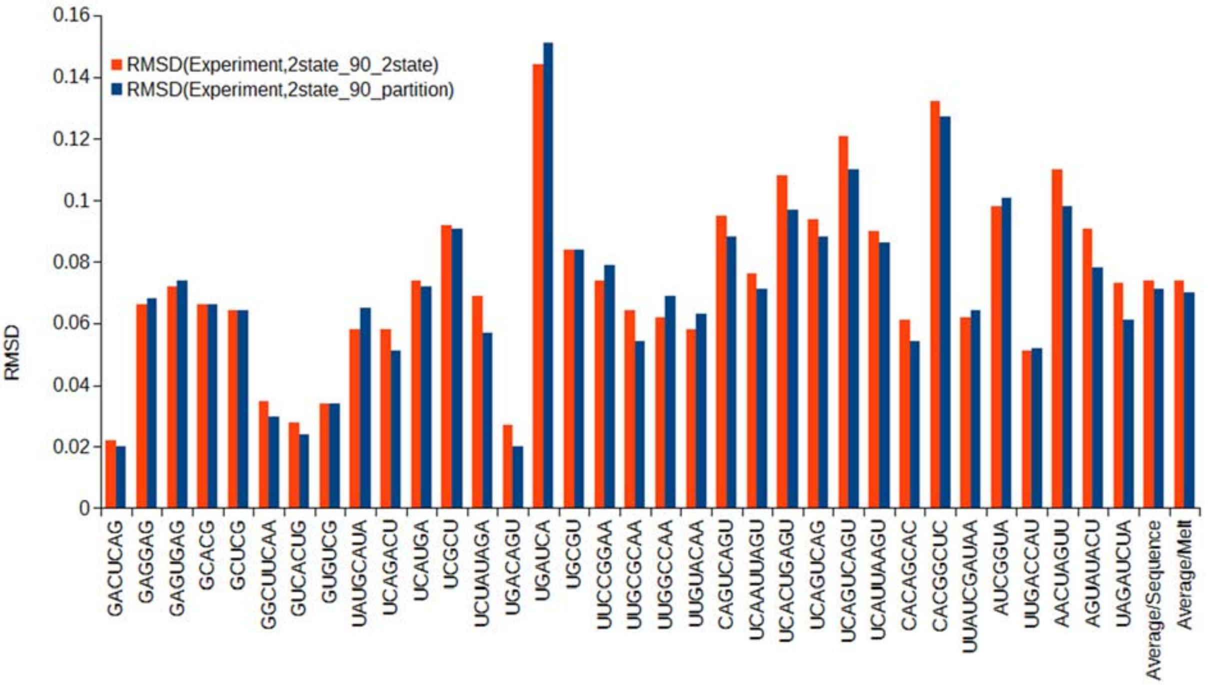

B:

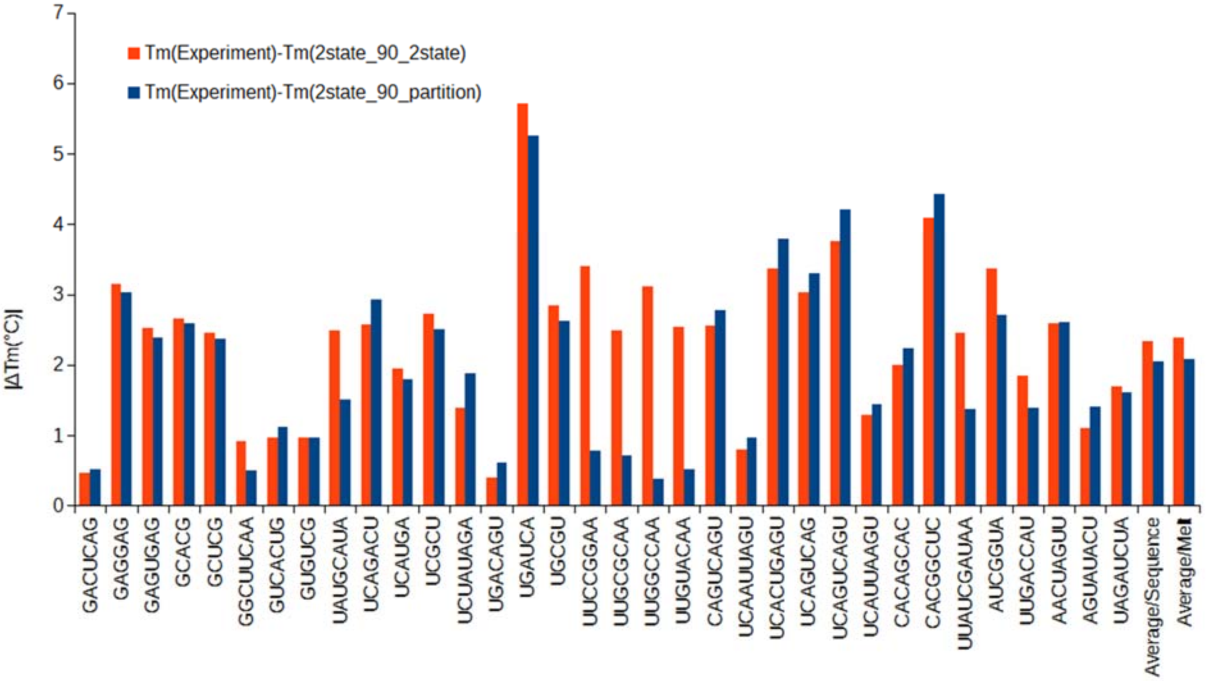

Figure S6. Comparison of root mean square deviations (RMSD) between experimental and estimated optical melting curves (panel A) and comparison of absolute values of differences between predicted melting temperatures and measured melting temperatures (panel B). All predictions were made using the current values of nearest neighbor parameters. Blue bars correspond to predictions made using the two-state model and the red bars are predictions made using partition function approach. First 34 bars

are averages over melts of 34 duplexes whose data was used here. Last two bars are averages per unique sequence and averages per melt.

REFERENCES

1. Bloomfield,V.A., Crothers,D.M. and Tinoco,I. (2000) Nucleic acids: structures, properties, and functions University Science Books, Sausalito, Calif.
